# Brain ventricle and choroid plexus morphology as predictor of treatment response in major depression: Findings from the EMBARC study

**DOI:** 10.1016/j.bbih.2023.100717

**Published:** 2023-12-20

**Authors:** Harald Murck, Maurizio Fava, Cristina Cusin, Cherise Chin Fatt, Madhukar Trivedi

**Affiliations:** a Dept. of Psychiatry and Psychotherapy, Philipps-University Marburg, Marburg, Germany; b Department of Psychiatry, Massachusetts General Hospital, Harvard Medical School, Boston, MA, USA; c The University of Texas Southwestern Medical Center, Department of Psychiatry, Center for Depression Research and Clinical Care, Dallas, USA

**Keywords:** therapy refractory depression, brain imaging, ventricular volume, choroid plexu, corpus callosum, triglycerides, inflammation, prognostic biomarker

## Abstract

Recent observations suggest a role of the volume of the cerebral ventricle volume, corpus callosum (CC) segment volume, in particular that of the central-anterior part, and choroid plexus (CP) volume for treatment resistance of major depressive disorder (MDD). An increased CP volume has been associated with increased inflammatory activity and changes in the structure of the ventricles and corpus callosum. We attempt to replicate and confirm that these imaging markers are associated with clinical outcome in subjects from the EMBARC study, as implied by a recent pilot study. The EMBARC study is a placebo controlled randomized study comparing sertraline vs. placebo in patients with MDD to identify biological markers of therapy resistance. Association of baseline volumes of the lateral ventricles (LVV), choroid plexus volume (CPV) and volume of segments of the CC with treatment response after 4 weeks treatment was evaluated. 171 subjects (61 male, 110 female) completed the 4 week assessments; gender and age were taken into account for this analyses. As previously reported, no treatment effect of sertraline vs. placebo was observed, therefore the study characterized prognostic markers of response in the pooled population. Change in depression severity was identified by the ratio of the Hamilton-Depression rating scale 17 (HAMD-17) at week 4 divided by the HAMD-17 at baseline (HAMD-17 ratio). Volumes of the lateral ventricles and choroid plexi were positively correlated with the HAMD-17 ratio, indication worse outcome with larger ventricles and choroid plexus volumes, whereas the volume of the central-anterior corpus callosum was negatively correlated with the HAMD-17 ratio. Responders (n = 54) had significantly smaller volumes of the lateral ventricles and CP compared to non-responders (n = 117), whereas the volume of mid-anterior CC was significantly larger compared to non-responders (n = 117), confirming our previous findings. In an exploratory way associations between enlarged LVV and CPV and signs of lipid dysregulation were observed. In conclusion, we confirmed that volumes of lateral ventricles, choroid plexi and the mid-anterior corpus callosum are associated with clinical improvement of depression and may be indicators of metabolic/inflammatory activity.

## Highlights

• Easily accessible imaging markers predict worse outcome in major depression:
• Increased choroid plexus volume
• Increased lateral ventricle volume
• Reduced volume of central corpus callosum segments
• Choroid plexus may mediate inflammatory processes to affect mood regulation.

## 1 Introduction

The pathophysiology of major depressive disorder (MDD) is heterogeneous. The identification of effective compounds on the basis of a specific underlying neurobiology is hampered by the currently accepted definition of MDD in relevant classifications, including the DSM-5, which does not take biological differentiation into account. Importantly, this variability may not only affect the response to a given pharmacotherapy, but also the natural course of clinical change. This situation has negative implications in the context of clinical trials: Treatment arms are compared, which may show neurobiological heterogeneity at baseline. Patient stratification on the basis of biological variables may be able to define biological subtypes with a better chance to show a treatment effect in a targeted population.

Markers, which differentiate patients with depression into more biologically homogeneous subgroups have been reported. These include inflammatory markers (Dantzer et al., 2008; Lamers et al., 2013; Miller et al., 2009; Raison et al., 2013; Richter et al., 2010), metabolic markers, in particular those related to metabolic syndrome (Lamers et al., 2010; Richter et al., 2010), and neuroendocrine (Juruena et al., 2010; Lamers et al., 2013; Murck et al., 2012) characteristics. More recently, markers of autonomic regulation, including blood pressure and heart rate variability received renewed attention (Buttner et al., 2015; Engelmann et al., 2022; Kemp et al., 2010; Licht et al., 2008). These markers may have prognostic or predictive value in clinical trials and clinical practice.

Furthermore, imaging biomarkers have been characterized which predict clinical response and which may be useful to define biological subtypes. Many of these, including volumetry of gray- or white matter segments, are of high importance from a research perspective (Drevets et al., 1998; Samann et al., 2013), but are difficult to assess in standard practice. From a practical perspective a more easily accessible imaging marker, which is unfortunately frequently not reported in recent imaging studies, is cerebral lateral ventricular volume (LVV). LVV was frequently assessed already in the pre-MRI era by means of computer tomography. In the context of depression, LVV is increased in patients with depression in comparison to healthy controls (Kempton et al., 2011; Schlegel et al., 1989; Via et al., 2012) and was related to treatment outcome in one early study (Cardoner et al., 2003). It is often regarded as biologically unspecific, based on volume changes in multiple gray and white matter areas, but this may not completely explain observed changes of this parameter.

Here we explore the alternative hypothesis that choroid plexus drives ventricular expansion, which results in the compression of surrounding anatomical areas, in particular parts of the corpus callosum. We recently demonstrated an association between an increased LVV and CPV and worse treatment outcome in hospitalized patients with depression and identified moderators of this relationship (Murck et al., 2020), i.e. body mass index (BMI) and the salivary aldosterone/cortisol ratio. In this study we also observed a volume reduction of the mid-anterior and central corpus callosum segment volume in these patients, which was reciprocally correlated to LVV. This may indicate a compression of these corpus callosum segments, which could affect anatomical projection areas. In this context it is important to consider that LVV and CCV show structural plasticity in a reciprocal manner. Both are sensitive to sleep (Bernardi et al., 2016) and LVV is sensitive to stress, at least in animals (Henckens et al., 2015). A plausible mediator of this plasticity is the change in activity of the choroid plexus.

The volumetric determination of the CP is a relatively new area of investigation but is feasible with current MRI techniques. Changes have been described in complex pain syndrome (Zhou et al., 2015), anorexia nervosa (Lavagnino et al., 2015), multiple sclerosis (Fleischer et al., 2021) and most recently in major depression (Althubaity et al., 2022; Bravi et al., 2023) and psychosis (Lizano et al., 2019; Senay et al., 2023). The publications of Althubaity et al. and Bravi et al. are of particular interest, as they show the association between inflammatory markers and choroid plexus volume in mood disorders.

The functional plasticity of this system is also indicated by the fact that stress leads to changes in gene expression of the CP in animal models. Stress-affected genes are those of receptors, which have been linked to major depressive disorder already (MDD), including 5-HT2a, 5-HT2c, glucocorticoid, TNFα, IL1β, BDNF (Sathyanesan et al., 2012) as well as IL1 receptor (Wong and Licinio, 1994) and the CRH-receptor (Wong et al., 1994). In addition, a reduced expression of pathways centered around transforming growth factor beta (TGFbeta) in the choroid plexus has also been reported in depression (Turner et al., 2014). TGFbeta acts as an anti-inflammatory mediator in the brain (Bajramovic, 2011) by inhibiting the TLR4-pathway (Naiki et al., 2005). In line with the reported findings, the volume of the CP has been proposed as a marker for inflammatory activity in the CNS (Turkheimer et al., 2023) and may not only reflect, but possibly mediate inflammatory and neuroendocrine changes in depression (Dantzer et al., 2008).

In summary, downstream mechanisms of the involvement of the CP are at least threefold: an increased CSF release may lead to a mechanical compression of anatomical areas, which are adjacent to the ventricles (Murck et al., 2022). Secondly, molecular moderators may spread from the CP into brain tissue via volume transmission (Dantzer, 2001; Skipor and Thiery, 2008). Those moderators may be produced by the CP itself or stem from the circulation. Finally, a reduction of the permeability of the blood brain barrier and the CP can be observed in conditions of inflammation (Carloni et al., 2021; Turkheimer et al., 2021). However, it is important to consider that the observed volume reduction of the corpus callosum may be a direct effect of inflammation (Cyprien et al., 2019), which may affect both choroid plexi and the corpus callosum in parallel.

We want to replicate our earlier findings of the relationship of clinical outcome with ventricular-, choroid plexus- and mid-anterior and central corpus callosum volume in a larger sample of patients with depression in this retrospective analysis from data from the EMBARC study. In an exploratory way we also correlate metabolic and autonomic markers with the volume of these anatomical areas in order to generate hypothesis about the causality of the observed relationships.

## 2 Methods

The EMBARC study characterized biological markers in a randomized placebo-controlled trial of sertraline vs. placebo in patients with MDD of 8 weeks duration, followed by an additional treatment section, based on the outcome of the first 8 weeks of treatment. For consort statement see (Cooper et al., 2019). This trial is conducted according to the Declaration of Helsinki. It was approved by the Institutional Review Board at each clinical site. Signed informed consent was obtained from subjects in order for participation in the trial. The main objective was to identify clinical and biological moderators of treatment response (Trivedi et al., 2016). Patients with early onset (before age 30), chronicity (episode duration >2 years) or recurrent MDD (two or more recurrences including current episode) were enrolled.

The clinical parameter of interest was the Hamilton-depression rating scale (17 item; HAMD-17). For correlational analysis of clinical improvement, we used the ratio between the HAMD-17 at outcome divided to the HAMD-17 at baseline (HAMD-17 ratio). A value of 1 means no change from baseline, a value of 0.7 means a reduction to 70% of the baseline value. Response was defined as a HAMD-17 ratio ≤0.5.

For these primary analyses we prospectively focused on subjects who completed the first 4 weeks of the placebo-controlled treatment phase. In the current dataset, 207 subjects had imaging and assessments with the Hamilton depression rating scale (HAMD) at baseline, of which 171 (age 37.5 ± 13.4; HAMD-17: 18.8 ± 4.7) had both assessments at week 4 as well. Age differed slightly, but significantly, between responders and non-responders (36.6 ± 11.6 vs. 37.2 ± 13.8 years, respectively (p < 0.05). We a priori chose the 4-week treatment interval in order to optimize the time for clinical improvement with the number of drop-outs. For a time course of the correlation of the HAMD-17 value with imaging parameters, which was generated as a sensitivity analysis and to show consistency, please see Table S1.

Imaging was processed as described before (Bartlett et al., 2018; Pillai et al., 2019; Trivedi et al., 2016). For details see (Bartlett et al., 2018). In short, MRI scans were performed at pretreatment. MRI scanning took place across 5 sites: University of Texas Southwestern Medical Center (TX—Philips Achieva, 8-channel head-coil), University of Michigan (UM—Philips Ingenia, 15-channel), Massachusetts General Hospital (MGH—Siemens TrioTim, 12-channel), Columbia University Medical Center (CU—GE Signa HDx, 8-channel), and Stony Brook Medical Center (SBU—Siemens TrioTim, 12-channel). T1-weighted image acquisition and test–retest reliability of processing have been published (Iscan et al., 2015). In short, MPRAGE sequences were acquired at TX, UM, MGH, and SBU, while an IR-FSPGR sequence was acquired at CU. Sequence parameters were as follows: TR/TE = 5.9–8.2/2.4–4.6 ms, 8–12° flip angle, 1 mm slice thickness, 4.4–5.5 min acquisition, and 1 mm isotropic voxel dimensions. Raw structural images were passed through Freesurfer 5.3.0 (http://surfer.nmr.mgh.harvard.edu/) to extract regions of interest. Freesurfer surface models underwent systematic quality control and for reliability between different sites (Iscan et al., 2015). Imaging processing included adjustments for potential site differences, which made a statistical factor of the site unnecessary (Bartlett et al., 2018).

Of the 171 subjects with imaging measures at baseline and 4 weeks associations of volumes of lateral ventricles (LVV), choroid plexus (CPV) and of the corpus callosum (CCV), with treatment response were evaluated. For the correlational analysis adjusted volumes were used, which took the total brain volume into account, i.e. volume of interest/total brain volume-ratio. The relationship of LVV, CPV and the volume of the mid-anterior and central part of the corpus callosum at baseline, and clinical changes as assessed with the HAMD-17 ratio were examined. Other CC segments are also reported but did not show a signal in the pilots study and therefore are regarded as exploratory. For the analysis of correlations Pearson correlation coefficients and p-values are provided. The correlations between outcome (HAMD-17 ratio at week 4) and the adjusted LVV, CPV, mid-anterior and central CC segment volumes are regarded as primary analysis, as these analyses served to replicate and potentially confirm our earlier findings. For those parameters, which showed a significant correlation, a regression analysis between the volume of interest and the HAMD-17 ratio at week 4 was performed, which took total brain volume, age and gender into account. From these regressions we also derived residuals in order to test for normality. Furthermore, response after 4 weeks from baseline (≤50 % reduction of the HAMD) was assessed to compare responders from non-responders; gender, age, and total brain volume were taken into account for the ANCOVA analyses. Due to the imbalance of the number of responders and non-responders (see below), this analysis mainly serves to ensure consistency with the correlational data. The correlations with metabolic and autonomic parameters have to be regarded as exploratory.

## 3 Results

The demographic, clinical and selected imaging characteristics of the population at baseline are presented in Table 1. A correlation analysis between baseline HAMD-17 and the volumes of interest was performed in order to determine potential state related associations. Choroid plexus volumes were significantly correlated with the HAMD-17 score at baseline (n = 217; right: Pearson R = 0.22, p = 0.002; left: Pearson R = 0.17, p = 0.017), whereas no correlation between ventricular volumes or corpus callosum sections and baseline depression severity could be detected (all p > 0.20 with the exception of the right lateral ventricle, which showed a trend toward a significant correlation (Pearson R = 0.13; p = 0.06)).

**Table 1:** **Descriptive Statistics of baseline characteristics and HAMD-17 over time** (HAMD: Hamilton depression rating scale; BMI, body mass index; CC: corpus callosum).

	n	Minimum	Maximum	Mean	SD
HAMD-17 baseline	207	7	32	18.8	4.5
HAMD-17 week 4	171	0	31	13.1	6.9
age	206	18	65	36.2	13.0
BMI	184	15.4	50.5	27.5	6.5
Volume right lateral ventricle	207	982.7	25820.4	6449.7	4073.7
Volume left lateral ventricle	207	1996.8	37401.8	7177.2	4619.8
Volume right choroid plexus	207	376.9	4107.6	1381.2	478.0
Volume left choroid plexus	207	332.3	3682.8	1208.2	388.4
Volume CC anterior	207	549.8	1331.1	861.8	140.4
Volume CC central	207	241.2	849.9	473.1	92.8
Volume CC mid-anterior	207	270.1	1231.6	474.6	112.6
Volume CC mid-posterior	207	229.4	715.1	438.8	84.7
Volume CC posterior	207	570.9	1302.4	948.2	132.2

**Fig. 1:**
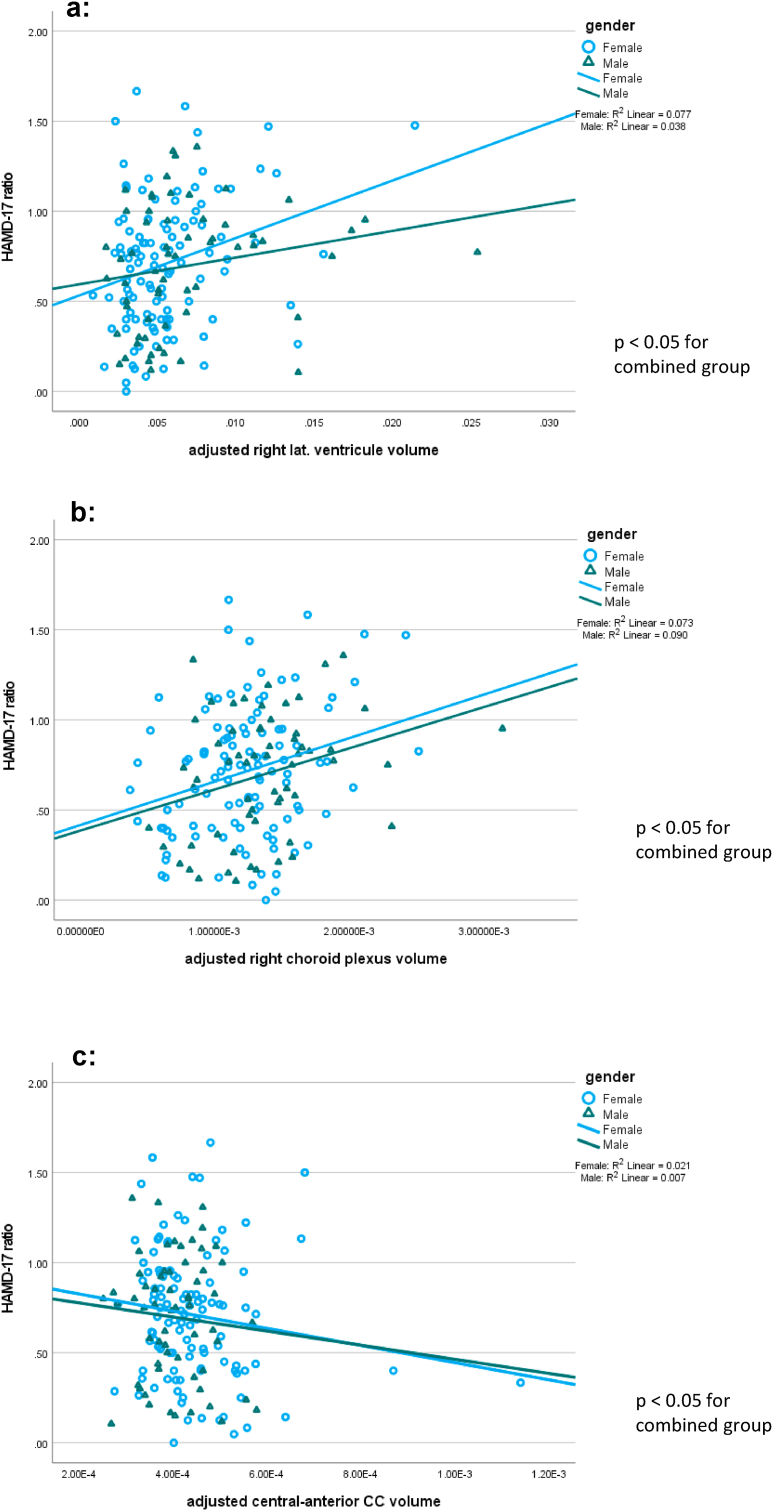
**Association between clinical outcome and morphological features**: a) Correlation of HAMD-17-ratio by right lateral ventricle volume (expressed as ratio of ventricular volume to total brain volume): a larger ventricle volume is associated with less favorable clinical improvement after 4 weeks, independent of gender. b: Correlation of HAMD-17-ratio with the right choroid plexus volume (as expressed as ratio between choroid plexus volume total brain volume): A large choroid plexus volume is associated with less favorable clinical improvement after 4 weeks, independent of gender. c: Correlation of HAMD-17-ratio by central anterior CC volume (as expressed as ratio between central anterior CC volume and total brain volume): A smaller CC volume is associated with less favorable clinical improvement after 4 weeks, independent of gender.

A Pearson correlation was used with HAMD-17 ratio at week 4 as outcome variable and the volumes of the selected anatomical areas. Scatterplots for the adjusted volumes are depicted in Fig. 1. These correlational analyses confirmed the relationship between clinical change and ventricular volume, choroid plexus volume and mid anterior CC segment volume at baseline., see Table S1 for details. The linear regression model with sex, age and intracerebral volume as covariates led to similar results and did not change the significance levels. For these regression analyses the residuals were normally distributed in all cases that showed a significant correlation. However, a correlation between the central CC volume and outcome could not be confirmed.

Comparing responders and non-responders, we first used a multivariate test for selected volumes, which were adjusted for gender, age and intracranial volume. An overall global significant difference between responders and non-responders was observed for volumetric parameters (p = 0.007, see Table 2). Univariate analyses revealed that responders at week 4 had significantly smaller volumes of the choroid plexi and lateral ventricles, whereas the volume of mid-anterior and mid-posterior CC was significantly larger compared to non-responders (Table 2). Due to the imbalance of the numbers of subjects in the responders vs. non-responders these results have to be taken with caution.

**Table 2:** Comparison of volume of anatomical structures in responders (≥50 % reduction of HAMD from baseline at week 4) vs. non-responders.

	Non-Responder (n = 117)		Responder (n = 54)		df	Mean Square	F	Sig.
Volumes (mm^3^)	Mean	SD	Mean	SD				
right lateral vent.	7118.6	4547.2	5916.9	3523.7	1	69683320	**4.630**	**.033**
left lateral vent.	7890.2	5317.6	6527.3	3510.0	1	90042251	**4.517**	**.035**
right CP	1471.9	497.1	1244.8	436.5	1	2044360	**10.631**	**.001**
left CP	1284.5	411.1	1109.0	330.4	1	1051920	**8.317**	**.004**
CC anterior	860.6	137.0	880.8	168.7	1	1398	.087	.769
CC mid-anterior	458.0	87.9	512.6	153.4	1	54259	**4.846**	**.029**
CC central	464.5	89.3	481.6	84.0	1	1482	.236	.628
CC mid-posterior	424.8	82.4	468.9	87.1	1	47513	**7.843**	**.006**
CC posterior	948.2	128.2	956.3	156.1	1	5084	.318	.573

**Fig. 2:**
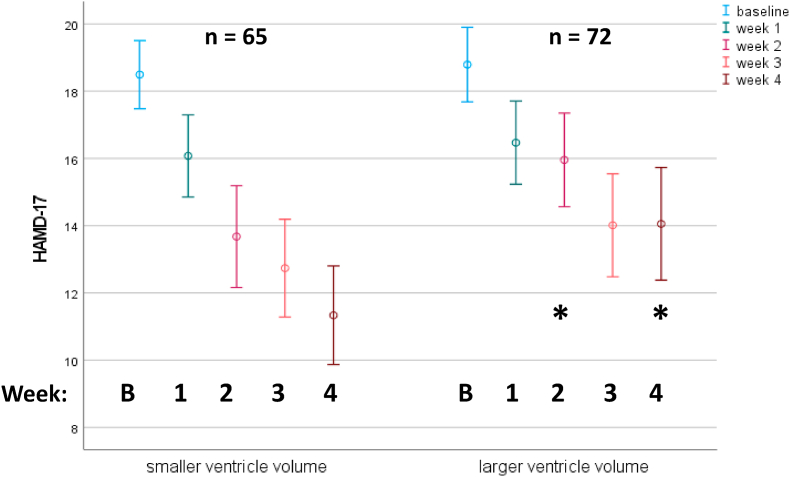
**Time course of HAMD-17 score in subjects with larger vs. smaller right lateral ventricle volume**. A median split of the right lateral ventricle volume was used to separate the groups. Only subjects without missing values are depicted.

Vice versa, splitting the population at the median for the right ventricular volume demonstrates the difference of the course of depressive symptoms between the two LVV groups clearly (Fig. 2): A significant difference between HAMD-17 scores for the high vs. low LVV-volume groups were observed at week 2 and week 4.

As a sensitivity analysis we also correlated the baseline volume of the anatomical structures with the relative outcome of the HAMD-17 (HAMD-ratio) over time. Both choroid plexus volumes at baseline determined clinical outcome at week 4, week 6 and week 8 (p < 0.05). For CC segment volumes (mid anterior and mid posterior) a statistically significant negative correlation was observed at week 3 and 4; For both left and right LVV a statistically significant positive correlation occurred at week 4, indicating worse outcome with larger ventricles. Please see Suppl. Table S1 for the stability of the correlation between volume of anatomical structures and treatment effect over time.

In addition, we explored factors which may affect the volume of the anatomical areas in an exploratory fashion at baseline (Table 3). We found that the volumes of both lateral ventricles were positively correlated to LDL-cholesterol and triglyceride levels. The volume of the left LVV was significantly correlated to systolic blood pressure. A similar pattern was observed for the CP volumes. All these parameters are positively correlated to age, which we corrected for in the primary analyses.

**Table 3:** Correlation of volume of anatomical structures with of relative improvement, metabolic parameters and age.

		HAMD-17 ratio (N = 171)	Syst BP (n = 195)	Weight (n = 192)	Height (n = 184)	BMI (n = 184)	LDL-Chol. (n = 184)	Trigl. (n = 190)	age (n = 206)
right lateral vent.	Pearson	**.198**	.123	.131	.106	.094	**.176**	**.290**	**.313**
	Sig.	**.009**	.086	.069	.154	.206	**.017**	**.000**	**.000**
left lateral vent.	Pearson	**.177**	**.191**	**.219**	**.204**	**.176**	**.182**	**.216**	**.259**
	Sig.	**.021**	**.008**	**.002**	**.005**	**.017**	**.013**	**.003**	**.000**
right CP	Pearson	**.239**	**.229**	.141	**.251**	.052	.122	**.233**	**.276**
	Sig.	**.002**	**.001**	.050	**<.001**	.482	.098	**.001**	**.000**
left CP	Pearson	**.222**	**.158**	.099	**.225**	.023	.105	**.253**	**.194**
	Sig.	**.004**	**.027**	.173	**.002**	.757	.155	**.000**	**.005**
CC anterior	Pearson	−.044	.019	.107	**.203**	.010	.007	−.083	−.051
	Sig.	.564	.791	.138	**.006**	.889	.924	.258	.468
CC mid-anterior	Pearson	**−.160**	.000	.111	.053	.096	−.053	−.014	−.107
	Sig.	**.037**	.996	.124	.473	.195	.478	.851	.126
CC central	Pearson	−.063	−.074	.027	.111	−.037	−.140	−.092	**−.168**
	Sig.	.411	.305	.714	.134	.620	.058	.208	**.016**
CC mid-posterior	Pearson	**−.219**	−.103	.017	**.168**	−.071	−.087	−.095	**−.171**
	Sig.	**.004**	.152	.819	**.023**	.341	.241	.191	**.014**
CC posterior	Pearson	.042	.059	**.165**	**.169**	.093	.013	.008	.058
	Sig.	.581	.414	**.022**	**.021**	.211	.857	.912	.410

In order to determine the relationship between the anatomical areas of interest, ventricular volumes were correlated with CC segment- and CP volumes. A significant positive correlation between CP volumes and lateral ventricle volumes was established. Furthermore, a significant negative correlation between third ventricular volumes and the mid-anterior and mid-posterior CC segments, as well as a significant negative correlation between the lateral ventricles and the central and mid-posterior CC volume were observed (Table S2).

DTI parameters were assessed for the corpus callosum and did not predict outcome. However, the volume of the mid-anterior and mid posterior CC segments, adjusted for total brain volume, correlated negatively with the axial diffusivity of these segments (mid-anterior: R = −0.42, p < 0.001, n = 191; mid-posterior: R = −0.15; p = 0.036, n = 196), whereas the CC-segment volumes were not associated with fractional anisotropy (for all, p > 0.1).

## 4 Discussion

The outcome of this study is twofold, one more practical, others theoretical: Firstly, an easily accessible imaging marker, i.e. lateral ventricular volume, shows a strong predictive value for the improvement of depressive symptoms in MDD patients treated with either sertraline or placebo. From a theoretical, mechanistical aspect the strong relationship of ventricular volume to both choroid plexus volume and the volume of selected CC segments could be of interest for the pathophysiology of some forms of MDD. A working hypothesis is that changes in choroid plexus function, i.e. an increased release of CSF (Murck et al., 2020; Sheldon et al., 2015), or an increased release of specific bioactive molecules, including inflammation mediators (Dantzer, 2001; Fleischer et al., 2021) may lead to a change in white matter volume and/or integrity. This can be tested in prospective studies. Increased ventricular pressure or, alternatively, such bioactive molecules may affect white matter function either by mechanical compression or by an effect on white matter integrity via alternations of oligodendrocyte function. This could be related to changes in myelination or changes in the volume regulation of axons within the CC. Indeed: disturbance of white matter integrity has frequently been described in patients with depressive disorders, mainly by using diffusion tensor imaging (DTI) methods (Benedetti et al., 2011; Chen et al., 2017; de Diego-Adelino et al., 2014; Guo et al., 2012b; Repple et al., 2017; Wise et al., 2016). In addition, a direct effect on corpus callosum integrity for example via inflammatory or metabolic/energy related mechanisms is a possibility, which needs further exploration (Bartzokis, 2011; Connor and Menzies, 1996; Murck et al., 2022). In line with this notion a negative correlation between C-reactive protein concentration and anterior and mid-corpus callosum volume has been demonstrated in elderly patients with depression (Cyprien et al., 2019). It would be of interest to study, whether anti-inflammatory compounds lead to changes in choroid plexus-, ventricular- and corpus callosum volume and if these changes are related or occur independently and whether these are associated with clinical improvement.

Support of involvement of the choroid plexus in mood disorders was reported recently (Althubaity et al., 2022; Bravi et al., 2023) and linked to inflammatory activity and changes in blood brain barrier permeability (Althubaity et al., 2022; Bravi et al., 2023; Cao et al., 2023).

Besides inflammatory mechanisms, the volume of the choroid plexus is affected by neuroendocrine influences, which have been linked to MDD, in particular vasopressin and aldosterone (Murck et al., 2020; Sheldon et al., 2015), as well as metabolic parameters related to an increased BMI. Therefore, activation of inflammatory processes appears to be a plausible, but not exclusive explanation (Baumeister et al., 2016; Coelho et al., 2014; Nemeroff, 2016). In particular, high fat diet is related to an increased ventricular volume in animals in the context of traumatic stress (Kalyan-Masih et al., 2016). Similarly, a link between increased ventricular volume and hyperlipidemia has been observed in subjects with normal pressure hydrocephalus (Israelsson et al., 2017). In line with this observation, our pilot study identified a strong correlation between BMI and both choroid plexus- and ventricular volume (Murck et al., 2020). The finding of the current study of the correlation between triglyceride levels at baseline with choroid plexus volumes and ventricular volumes reported here confirm the influence of metabolic parameters on brain morphology and together may be useful to differentiate patients with depression to define more homogeneous biological subgroups, as reported earlier (Lamers et al., 2013). It is therefore of interest to study the effects of interventions, which affect energy metabolism, including exercise, fasting, or lipid lowering compounds in the choroid plexus volume and how its potential change relates to clinical outcome (Murck et al., 2020; Nilsson et al., 1992). Interestingly, these findings are also in line with the role of inflammation as both aldosterone (Bay-Richter et al., 2012; Hlavacova et al., 2012; Rocha et al., 2002) and dyslipidemia and increased BMI (Cooper et al., 2012; de Kloet et al., 2014; Miller et al., 2003; Pires et al., 2018) show a close association to increased inflammation.

Our observation that the HAMD-17 score at baseline correlates significantly to choroid plexus volumes, but only by trend to ventricular volumes and not to CC segment volumes, implies that choroid plexus volume shows a state like characteristic, whereas ventricular volume may have a more trait/chronicity related characteristic. Corpus callosum segments volume appears mainly to be a trait- or risk marker. As mentioned in the introduction, stress leads to an increase in ventricular volume in animals (Henckens et al., 2015). Childhood abuse has been related to an increase in ventricular volumes and reduced white matter volume in later life (De Bellis et al., 1999; De Bellis and Zisk, 2014), to therapy refractoriness in depression (Heim and Nemeroff, 2001; Nelson et al., 2017) and increased inflammatory activity (Baumeister et al., 2016; Lippard and Nemeroff, 2022).

No association between DTI parameters with clinical change was observed. This is in contrast to studies, which reported DTI parameters as predictive for response, for example to ketamine (Sydnor et al., 2020; Vasavada et al., 2016). Nevertheless, we observed that the volume of CC segments correlated inversely with axial diffusivity (AD): a smaller CC segment volume was correlated to an increased AD. An earlier DTI report from the EMBARC study, which focused on the structural connectivity in specific anatomical areas did find an increase in fractional anisotropy (FA) in non-remitters (Pillai et al., 2019). As AD and FA are correlated, this outcome appears consistent, but is nevertheless in contrast to a number of other studies (Chen et al., 2017; de Diego-Adelino et al., 2014; Guo et al., 2012a; Repple et al., 2017; Wise et al., 2016). This shows the importance to take into consideration that FA and AD and other DTI markers can be influenced by varying mechanisms, which depend on the structural integrity of an axon, but also on axonal density (Bishop et al., 2018). The reporting of the volume of white matter structures in the context of DTI studies may help to clarify these discrepancies.

Limitations of the study are the post hoc nature of the current analyses, however, they were motivated by the attempt to replicate data from an earlier study (Murck et al., 2020) and the primary variables of interest are identical to the identified parameters from the pilot study. Therefore, with all caution, the current analysis overall confirms the results of our previous pilot study. It has, however, to be considered that inclusion/exclusion criteria differ between the studies. Regarding the imaging methodology, whereas the volume determination of the corpus callosum and ventricles appears well established, the measurement of choroid plexus volume has an exploratory element. A correlation between freesurfer-established volumes and manually performed segmentation has been demonstrated (Lizano et al., 2019), but variability was recognized. However, an automated segmentation with a visual quality control, as in our study, appears to be acceptable in trials with larger numbers of subjects (Bravi et al., 2023). Better methods for the segmentation of the choroid plexus are nevertheless a high priority (Bannai et al., 2020).

In conclusion, we (re-)identified an easily accessible imaging marker which appears to be related to the clinical course of depression. Ventricular volume may affect other imaging parameters and should therefore be taken into account in future imaging studies, at least in studies in MDD. In addition, the current findings go beyond a strictly descriptive association. With the additional observation of the relationship of increased ventricular volumes and increased choroid plexus volumes, our findings provide a plausible hypothesis, how neuroendocrine and metabolic parameters may mechanistically influence depressive symptoms. A new focus on choroid plexus function in stress-related disorders is supported, which, however, requires further methodological improvements. In the meantime, LVV, which is easily accessible, may provide a good correlate for practical purposes to identify subjects less responsive to therapy.

## Registration

ClinicalTrials.gov: Establishing Moderators and Biosignatures of Antidepressant Response for Clinical Care for Depression (EMBARC); https://clinicaltrials.gov/ct2/show/NCT01407094; NCT01407094.

## Authors contributions:

Maurizio Fava: Conceptualization, Funding acquisition, Investigation, Methodology, Supervision, Writing – review & editing. Harald Murck: Conceptualization, Formal analysis, Writing – original draft, Writing – review & editing. Cherise Chin Fatt: Conceptualization, Investigation, Writing – review & editing. Cristina Cusin: Conceptualization, Investigation, Writing – review & editing. Madhukar Trivedi: Conceptualization, Funding acquisition, Investigation, Methodology, Project administration, Supervision, Writing – review & editing

## Declaration of competing interest

The authors declare the following financial interests/personal relationships which may be considered as potential competing interests:

HM: is the owner of Murck-Neuroscience LLC, a consulting company, which also develops a patent in the area of major depression.

MF: lifetime disclosures: Research Support: Abbott Laboratories; Acadia Pharmaceuticals; Alkermes, Inc.; American Cyanamid; Aspect Medical Systems; AstraZeneca; Avanir Pharmaceuticals; AXSOME Therapeutics; BioClinica, Inc; Biohaven; BioResearch; BrainCells Inc.; Bristol-Myers Squibb; CeNeRx BioPharma; Centrexion Therapeutics Corporation; Cephalon; Cerecor; Clarus Funds; Clexio Biosciences; Clintara, LLC; Covance; Covidien; Eli Lilly and Company;EnVivo Pharmaceuticals, Inc.; Euthymics Bioscience, Inc.; Forest Pharmaceuticals, Inc.; FORUM Pharmaceuticals; Ganeden Biotech, Inc.; Gentelon, LLC; GlaxoSmithKline; Harvard Clinical Research Institute; Hoffman-LaRoche; Icon Clinical Research; Indivior; i3 Innovus/Ingenix; Janssen R&D, LLC; Jed Foundation; Johnson & Johnson Pharmaceutical Research & Development; Lichtwer Pharma GmbH; Lorex Pharmaceuticals; Lundbeck Inc.; Marinus Pharmaceuticals; MedAvante; Methylation Sciences Inc; National Alliance for Research on Schizophrenia & Depression (NARSAD); National Center for Complementary and Alternative Medicine (NCCAM); National Coordinating Center for Integrated Medicine (NiiCM); National Institute of Drug Abuse (NIDA); National Institutes of Health; National Institute of Mental Health (NIMH); Neuralstem, Inc.; NeuroRx; Novartis AG; Novaremed; Organon Pharmaceuticals; Otsuka Pharmaceutical Development, Inc.; PamLab, LLC.; Pfizer Inc.; Pharmacia-Upjohn; Pharmaceutical Research Associates., Inc.; Pharmavite® LLC; PharmoRx Therapeutics; Photothera; Praxis Precision Medicines; Premiere Research International; Protagenic Therapeutics, Inc.; Reckitt Benckiser; Relmada Therapeutics Inc.; Roche Pharmaceuticals; RCT Logic, LLC (formerly Clinical Trials Solutions, LLC); Sanofi-Aventis US LLC; Shenox Pharmaceuticals, LLC; Shire; Solvay Pharmaceuticals, Inc.; Stanley Medical Research Institute (SMRI); Synthelabo; Taisho Pharmaceuticals; Takeda Pharmaceuticals; Tal Medical; VistaGen; WinSanTor, Inc.; Wyeth- Ayerst Laboratories; Advisory Board/Consultant: Abbott Laboratories; Acadia; Aditum Bio Management Company, LLC; Affectis Pharmaceuticals AG; Alfasigma USA, Inc.; Alkermes, Inc.; Altimate Health Corporation; Amarin Pharma Inc.; Amorsa Therapeutics, Inc.; Ancora Bio, Inc.; Angelini S.p.A; Aptinyx Inc.; Arbor Pharmaceuticals, LLC; Aspect Medical Systems; Astella Pharma Global Development, Inc.; AstraZeneca; Auspex Pharmaceuticals; Avanir Pharmaceuticals; AXSOME Therapeutics; Bayer AG; Best Practice Project Management, Inc.; Biogen; BioMarin Pharmaceuticals, Inc.; BioXcel Therapeutics; Biovail Corporation; Boehringer Ingelheim; Boston Pharmaceuticals; BrainCells Inc; Bristol-Myers Squibb; Cambridge Science Corporation; CeNeRx BioPharma; Cephalon, Inc.; Cerecor; Clexio Biosciences; Click Therapeutics, Inc; CNS Response, Inc.; Compellis Pharmaceuticals; Cybin Corporation; Cypress Pharmaceutical, Inc.; DiagnoSearch Life Sciences (P) Ltd.; Dainippon Sumitomo Pharma Co. Inc.; Dr. Katz, Inc.; Dov Pharmaceuticals, Inc.; Edgemont Pharmaceuticals, Inc.; Eisai Inc.; Eli Lilly and Company; ElMindA; EnVivo Pharmaceuticals, Inc.; Enzymotec LTD; ePharmaSolutions; EPIX Pharmaceuticals, Inc.; Esthismos Research, Inc.; Euthymics Bioscience, Inc.; Evecxia Therapeutics, Inc.; ExpertConnect, LLC; FAAH Research Inc.; Fabre-Kramer Pharmaceuticals, Inc.; Forest Pharmaceuticals, Inc.; Forum Pharmaceuticals; Gate Neurosciences, Inc.; GenetikaPlus Ltd.; GenOmind, LLC; GlaxoSmithKline; Grunenthal GmbH; Happify; H. Lundbeck A/S; Indivior; i3 Innovus/Ingenis; Intracellular; Janssen Pharmaceutica; Jazz Pharmaceuticals, Inc.; JDS Therapeutics, LLC; Johnson & Johnson Pharmaceutical Research & Development, LLC; Knoll Pharmaceuticals Corp.; Labopharm Inc.; Lorex Pharmaceuticals; Lundbeck Inc.; Marinus Pharmaceuticals; MedAvante, Inc.; Merck & Co., Inc.; Mind Medicine Inc.; MSI Methylation Sciences, Inc.; Naurex, Inc.; Navitor Pharmaceuticals, Inc.; Nestle Health Sciences; Neuralstem, Inc.; Neurocrine Biosciences, Inc.; Neuronetics, Inc.; NextWave Pharmaceuticals; Niraxx Light Therapeutics, Inc; Northwestern University; Novartis AG; Nutrition 21; Opiant Pharmecuticals; Orexigen Therapeutics, Inc.; Organon

Pharmaceuticals; Osmotica; Otsuka Pharmaceuticals; Ovid Therapeutics, Inc.; Pamlab, LLC.; Perception Neuroscience; Pfizer Inc.; PharmaStar; PharmaTher Inc.; Pharmavite® LLC.; PharmoRx Therapeutics; Polaris Partners; Praxis Precision Medicines; Precision Human Biolaboratory; Prexa Pharmaceuticals, Inc.; Protagenic Therapeutics, Inc; PPD; PThera, LLC; Purdue Pharma; Puretech Ventures; Pure Tech LYT, Inc.; PsychoGenics; Psylin Neurosciences, Inc.; RCT Logic, LLC (formerly Clinical Trials Solutions, LLC); Relmada Therapeutics, Inc.; Rexahn Pharmaceuticals, Inc.; Ridge Diagnostics, Inc.; Roche; Sanofi-Aventis US LLC.; Sensorium Therapeutics; Sentier Therapeutics; Sepracor Inc.; Servier Laboratories; Schering-Plough Corporation; Shenox Pharmaceuticals, LLC; Solvay Pharmaceuticals, Inc.; Somaxon Pharmaceuticals, Inc.; Somerset Pharmaceuticals, Inc.; Sonde Health; Sunovion Pharmaceuticals; Supernus Pharmaceuticals, Inc.; Synthelabo; Taisho Pharmaceuticals; Takeda Pharmaceutical Company Limited; Tal Medical, Inc.; Tetragenex; Teva Pharmaceuticals; TransForm Pharmaceuticals, Inc.; Transcept Pharmaceuticals, Inc.; University of Michigan, Department of Psychiatry; Usona Institute,Inc.; Vanda Pharmaceuticals, Inc.; Versant Venture Management, LLC; VistaGen; Xenon Pharmaceuticals Inc.; Speaking/Publishing: Adamed, Co; Advanced Meeting Partners; American Psychiatric Association; American Society of Clinical Psychopharmacology; AstraZeneca; Belvoir Media Group; Boehringer Ingelheim GmbH; Bristol-Myers Squibb; Cephalon, Inc.; CME Institute/Physicians Postgraduate Press, Inc.; Eli Lilly and Company; Forest Pharmaceuticals, Inc.; GlaxoSmithKline; Global Medical Education, Inc.; Imedex, LLC; MGH Psychiatry Academy/Primedia; MGH Psychiatry Academy/Reed Elsevier; Novartis AG; Organon Pharmaceuticals; Pfizer Inc.; PharmaStar; United BioSource,Corp.; Wyeth-Ayerst Laboratories; Equity Holdings: Compellis; Neuromity; Psy Therapeutics; Sensorium Therapeutics; Royalty/patent, other income: Patents for Sequential Parallel Comparison Design (SPCD), licensed by MGH to Pharmaceutical Product Development, LLC (PPD) (US_7840419, US_7647235, US_7983936, US_8145504, US_8145505); and patent application for a combination of Ketamine plus Scopolamine in Major Depressive Disorder (MDD), licensed by MGH to Biohaven. Patents for pharmacogenomics of Depression Treatment with Folate (US_9546401, US_9540691). Copyright: for the MGH Cognitive & Physical Functioning Questionnaire (CPFQ), Sexual Functioning Inventory (SFI), Antidepressant Treatment Response Questionnaire (ATRQ), Discontinuation-Emergent Signs & Symptoms (DESS), Symptoms of Depression Questionnaire (SDQ), and SAFER; Belvoir; Lippincott, Williams & Wilkins; Wolkers Kluwer; World Scientific Publishing Co. Pte. Ltd.

CCF: nothing to disclose

CC: personal fees from Janssen, Perception, and Takeda; and grants from Clexio, Livanova, AFSP, and the National Institute of Mental Health.

MHT: research support from the Agency for Healthcare Research and Quality, Cyberonics Inc., National Alliance for Research in Schizophrenia and Depression, NIMH, National Institute on Drug Abuse, National Institute of Diabetes and Digestive and Kidney Diseases, and Johnson & Johnson; consulting and speaker fees from Abbott Laboratories Inc., Akzo (Organon Pharmaceuticals Inc.), Allergan Sales LLC, Alkermes, Astra Zeneca, Axon Advisors, Brintellix, Bristol-Myers Squibb Company, Cephalon Inc., Cerecor, Eli Lilly & Company, Evotec, Fabre Kramer Pharmaceuticals Inc., Forest Pharmaceuticals, GlaxoSmithKline, Health Research Associates, Johnson & Johnson, Lundbeck, MedAvante Medscape, Medtronic, Merck, Mitsubishi Tanabe Pharma Development America Inc., MSI Methylation Sciences Inc., Nestle Health Science-PamLab Inc., Naurex, Neuronetics, One Carbon Therapeutics Ltd, Otsuka Pharmaceuticals, Pamlab, Parke-Davis Pharmaceuticals Inc., Pfizer Inc., PgxHealth, Phoenix Marketing Solutions, Rexahn Pharmaceuticals, Ridge Diagnostics, Roche Products Ltd, Sepracor, SHIRE Development, Sierra, SK Life and Science, Sunovion, Takeda, Tal Medical/ Puretech Venture, Targacept, Transcept, VantagePoint, Vivus, and Wyeth- Ayerst Laboratories.

## Data Availability

Data will be made available on request.
