# Supplementary material for: Brain ventricle and choroid plexus morphology as predictor of treatment response in major depression: Findings from the EMBARC study
Source: Brain Behav Immun Health. 2023 Dec 20;35:100717. doi: 10.1016/j.bbih.2023.100717 (PMC10767278; doi:10.1016/j.bbih.2023.100717)
Supplement: Multimedia component 1 [file mmc1.rtf]

Murck et al, Brain Morphology and Treatment Response in Depression

Table S2: correlations of ventricle volumes with corpus callosum segment and choroid plexus volumes at baseline (n = 207). Bold numbers show significant correlations
	
	Volume third ventricle	Volume fourth ventricle	Volume right lateral ventricle	Volume left lateral ventricle	
Volume CC anterior	Pearson 	-.054	.018	.150	.204	
	Sig. (2-tailed)	.439	.796	.031	.003	
Volume CC mid-anterior	Pearson 	-.156	-.007	-.109	-.081	
	Sig. (2-tailed)	.025	.924	.118	.244	
Volume CC central	Pearson 	-.169	.020	-.225	-.241	
	Sig. (2-tailed)	.015	.773	.001	.000	
Volume CC mid-posterior	Pearson 	-.158	.015	-.255	-.277	
	Sig. (2-tailed)	.023	.831	.000	.000	
Volume CC posterior	Pearson 	.007	.026	.202	.230	
	Sig. (2-tailed)	.916	.712	.004	.001	
Volume right choroid plexus	Pearson 	.430	.277	.658	.678	
	Sig. (2-tailed)	.000	.000	.000	.000	
Volume left choroid plexus	Pearson 	.437	.265	.653	.698	
	Sig. (2-tailed)	.000	.000	.000	.000	
	
	
