# Supplementary material for: Brain ventricle and choroid plexus morphology as predictor of treatment response in major depression: Findings from the EMBARC study
Source: Brain Behav Immun Health. 2023 Dec 20;35:100717. doi: 10.1016/j.bbih.2023.100717 (PMC10767278; doi:10.1016/j.bbih.2023.100717)
Supplement: Multimedia component 2 [file mmc2.rtf]

Murck et al, Brain Morphology and Treatment Response in Depression

Supplemental table S1: Correlation of relative treatment outcome by week with volume of anatomical structures. Bold numbers show significant correlations.
	
	Week 1	Week 2	Week 3	Week 4	Week 6	Week 8	
	(n = 181)	(n = 165)	(n = 166)	(n = 171)	(n = 159)	(n = 158)	
right lateral vent.	Pearson	.059	.149	.096	.198	.130	.120	
	Sig. 	.434	.057	.217	.009	.103	.133	
left lateral vent.	Pearson 	-.008	.081	.040	.177	.095	.148	
	Sig. 	.913	.298	.612	.021	.233	.063	
right CP
	Pearson 	.087	.096	.110	.239	.193	.190	
	Sig. 	.244	.220	.160	.002	.015	.017	
left CP
	Pearson 	.063	.074	.139	.222	.157	.203	
	Sig. 	.401	.343	.073	.004	.048	.010	
CC anterior	Pearson 	-.029	-.056	-.099	-.044	-.102	-.002	
	Sig. 	.698	.473	.205	.564	.203	.982	
CC mid-anterior
	Pearson 	.045	-.018	-.154	-.160	-.104	-.051	
	Sig. 	.544	.814	.048	.037	.193	.528	
CC central	Pearson 	.002	-.114	-.045	-.063	-.122	-.014	
	Sig. 	.979	.144	.562	.411	.127	.858	
CC mid-posterior	Pearson 	-.035	-.094	-.164	-.219	-.139	-.044	
	Sig. 	.641	.229	.035	.004	.080	.580	
CC posterior	Pearson 	.026	.050	-.034	.042	.047	.144	
	Sig. 	.724	.521	.660	.581	.557	.072	
	
